# Physiologic model of the cerebrovascular system using supply and demand between arteries and tissues

**DOI:** 10.1038/s41598-025-10223-7

**Published:** 2025-07-30

**Authors:** Chang Min Lee, Hans Christian Rundfeldt, Keun-Hwa Jung, Hyeyeon Chang, Hyun Jin Kim

**Affiliations:** 1 Department of Mechanical Engineering, Korea Advanced Institute of Science and Technology, Daejeon, Republic of Korea; 2 Translational Neuroimaging Group, Center for Image Sciences, University Medical Center Utrecht, Utrecht, Netherlands; 3 Department of Neurology, Seoul National University Hospital, Seoul, Republic of Korea; 4 Department of Neurology, Konyang University Hospital, Daejeon, Republic of Korea

**Keywords:** Physiologic model, Cerebrovascular system, Supply and demand relationship, Perfusion territory, Blood flow Estimation of cerebral arteries, Boundary condition, Biomedical engineering, Mechanical engineering

## Abstract

Image-based modeling heavily relies on boundary conditions to obtain realistic blood flow and pressure. For the cerebrovascular system, boundary conditions are derived using in-vivo measurements or geometry-based models such as Murray’s law, but these are constrained by the image resolution or high sensitivity to the segmented geometry. We propose a physiologic model of the cerebrovascular system based on a supply and demand relationship between arteries and tissues. Blood flow and perfusion territory are determined by associating brain tissues with nearby vessels using Voronoi tessellation. The model was evaluated for 40 healthy young individuals and two diseased patients, and was validated by comparing the estimated blood flows and perfusion territories against literature data and perfusion imaging. The estimated blood flows are within the physiologically reported values for major cerebral arteries and the predicted perfusion territories are similar to the literature and perfusion imaging. Further, the model demonstrates more robustness to segmentation uncertainties compared to Murray’s law. The proposed model is shown to estimate physiologically plausible cerebrovascular blood flow and perfusion territory in a subject-specific manner using medical image data only. It may be used to simulate blood flow more realistically by developing boundary conditions based on this model in the cerebrovascular system.

**Supplementary Information:**

The online version contains supplementary material available at 10.1038/s41598-025-10223-7.

## Introduction

Numerous studies on image-based modeling of the cerebrovascular system have been conducted to non-invasively and realistically diagnose the severity of cerebrovascular diseases, predict outcomes of treatments, and design and evaluate medical devices^1–4^. To obtain reliable diagnostic and predictive modeling tools, however, it is critical to estimate blood flows realistically. Particularly in blood flow simulations, establishing boundary conditions that accurately represent the physical conditions of the cerebrovascular system beyond the modeled domain is crucial to obtain realistic results. Boundary conditions are enforced either strongly by prescribing flows or weakly by assigning pressure values or an implicit relationship between pressure and flow. For both cases, flow supplied to each boundary need to be estimated realistically to approximate the part of the cerebrovascular system not present in the modeled domain.

Various endeavors have been carried out to either measure or estimate blood flows of the cerebrovascular system non-invasively. PC-MRI^5^ or transcranial Doppler^6^ is a prevalent measurement modality for estimating blood flows of relatively large cerebral, internal carotid, and vertebral arteries. The measured flow rates can be directly applied as boundary conditions in blood flow simulations. The measurement techniques, however, are not reliable as the vessel caliber decreases due to the limitations in image resolution and artifacts. Recently, a study of estimating blood flows using perfusion imaging have been reported^7^. However, its application to the cerebrovascular system with an extensive vascular network of vessels poses a challenge.

When the flow measurement is not available, alternative approaches are taken to estimate boundary conditions. The simplest approach is to set the outlet pressure to zero or a constant value. This simple approach is utilized when simulating blood flows in the cerebral aneurysm models^8–10^. However, this method is applicable to single vessel models only as the flow distribution for multivessel models is not physiological. As an alternative, Murray’s law^11^ based flow estimation strategies have been widely reported^12,13^. Murray’s law is based on a physiologic principle that the vascular system is developed to minimize metabolic work and assumes that flow rate is proportional to the cube of the vessel radius. It is however highly sensitive to the segmented geometry as the vessel radius and the number of the segmented vessels nonlinearly affect blood flows based on the law. To compensate for the limitations of Murray’s law, various modified strategies have been developed^14,15^. In the splitting method, for example, the flow rate is locally divided based on the ratio of the radii of the daughter vessels at each bifurcation. While the modified strategies can reduce errors caused by inaccurately measured radii of outlets and missed branch vessels, they are still sensitive to the vessel radius depending on how the segmented geometry is constructed.

Lately, methods of estimating blood flows based on a supply and demand relationship between vessels and tissues have been developed for the coronary system^16^. Using these methods, tissues are associated with nearby vessels using a tessellation-based method. Unlike Murray’s law, which depends only on the segmented vascular geometry, this method uses both vascular and tissue geometries and may therefore be more accurate and robust to changes in vessel radius. This method has been demonstrated to be reliable in the coronary system by comparison with multiple in vivo and ex vivo flow measurements and perfusion imaging^16–18^. However, the methods have not been tested in the cerebrovascular system, which has several unique characteristics. First, the brain has a well-developed collateral circulation, which allows multiple vessels to supply blood to the same brain tissue^19^. This redundancy ensures that even if one vessel is compromised, other vessels can maintain adequate blood supply to the brain. Additionally, there are a number of perforating arteries that branch off directly from major blood vessels to supply blood to the central brain regions^20^.

In this study, we develop a physiologic model of the cerebrovascular system based on a supply and demand relationship between arteries and tissues. As the proposed model relies on the association between the arteries and tissues, the model is less sensitive to the segmented geometry and the perfusion territory of the cerebral arteries is obtained naturally through the process. To investigate the applicability of the model to actual patients, we first computed population-averaged blood flow rates and perfusion territories of major cerebrovascular arteries for 40 healthy young patients and compared the blood flow distribution and perfusion territories against those of the literature data. Further, we estimated perfusion territories of two diseased patients and compared with actual perfusion imaging. Finally, a sensitivity study was performed by varying a truncation radius value of the segmented geometry to evaluate the sensitivity of the proposed model to the vessel radius and the number of vessels of the segmented geometry.

## Materials and methods

### Construction of the cerebrovascular arteries and the brain tissue from medical image data

**Fig. 1:**
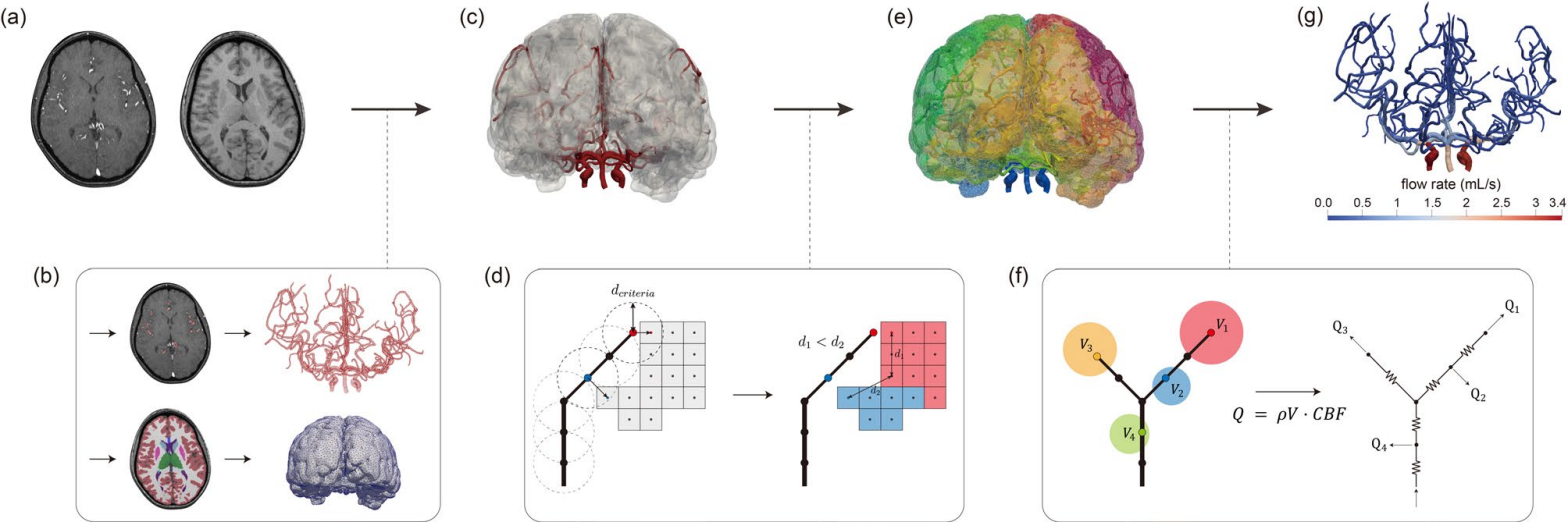
Overview of the processing pipeline for the physiologic model of the cerebrovascular system. (**a**) Input MRI and MRA images. (**b**) Perform segmentation of vessels and tissues from input images, followed by vessel centerline extraction and tissue mesh generation. (**c**) Align tissue mesh with the vascular network. (**d**) Select source points and perform Voronoi tessellation. (**e**) Estimate perfusion territories. (**f**) Compute blood flow rate required for perfusion of each region by multiplying the mass of the perfusion territory by CBF and distribute flows to the cerebrovascular arteries by solving one-dimensional blood flow simulations. (**g**) Report computed flow rates for all cerebrovascular arteries. MRA: MR angiography; CBF: cerebral blood flow.

The MR images shown in Fig. 1(a) were processed in the following steps. Vessel geometries were segmented as extensively as possible from the MR angiography using the three-dimensional (3D) growing region algorithm implemented in the Medical Imaging Interaction ToolKit^21^ (https://www.mitk.org/wiki/MITK, version 2022.04) as shown in Fig. 1(b). 3D Slicer (https://www.slicer.org, version 5.1.0) was then utilized to remove segmentation errors and separate vessels that merged by accident^22^. Lastly, vascular networks were extracted using the Vascular Modeling ToolKit (http://www.vmtk.org, version 1.4.0), with the spacing between the points in the vessel networks set to 0.5 mm^23^. The constructed vessel networks consist of points represented by the location of the vessel center points and the radius values, representing the geometry as a collection of thin, circular slabs between two consecutive points.

Tissue geometries were constructed using FreeSurfer^24^ (https://surfer.nmr.mgh.harvard.edu, version 7.4.1) from the T1-weighted MR image as shown in Fig. 1(b). The ventricles were removed from the geometries. A solid construction tool in Autodesk Meshmixer (https://www.autodesk.com, version 3.5.0) was used to fix a non-manifold geometry^25^and the volume mesh of the tissue was generated with the Computational Geometry Algorithm Library^26^ (https://www.cgal.org, version 5.4). Finally, the volume mesh was aligned with the vascular networks using the registration module of 3D Slicer as shown in Fig. 1(c)^27^.

### Physiologic model based on a supply and demand relationship

The proposed physiologic model utilizes vascular and tissue geometries constructed from medical image data. The vascular network consists of segments, defined as collections of points between two junction points, and the tissue geometry is discretized into tetrahedral elements.

Assuming that tissues are perfused by adjacent vessels based on a supply and demand relationship of arteries and tissues, Voronoi tessellation is used to estimate a perfusion territory for each artery as follows:

$$\:{R}_{i}=\:\left\{\:\frac{\parallel\:\overrightarrow{x}-{\overrightarrow{p}}_{i}\parallel\:}{\parallel\:{\overrightarrow{c}}_{i}\parallel\:}\le\:\:\frac{\parallel\:\overrightarrow{x}-{\overrightarrow{p}}_{j}\parallel\:}{\parallel\:{\overrightarrow{c}}_{j}\parallel\:},\:\:\forall\:j\ne\:i\:\:\right\}$$

Where $\:\overrightarrow{x}$ is a tissue location and $\:{\overrightarrow{p}}_{i}$ and $\:{\overrightarrow{p}}_{j}$ are positions of the $\:i$th and $\:j$th source points which belong to the segmented arteries, respectively. The tessellation propagation speeds of the $\:i$th and $\:j$th source points are represented by $\:{\overrightarrow{c}}_{i}$ and $\:{\overrightarrow{c}}_{j}$, respectively. The tessellation propagation speed can be set to be uniform or non-uniform depending on the blood flow. Further, it can be treated differently depending on whether the source is an outlet or a perforating artery. A uniform tessellation propagation speed is used for this study. To model perfusion through perforating arteries which are abundant in the cerebral vasculature, all points in the vascular network within a distance of 3 mm to the nearest tissue element were set as source points in addition to the vessel outlets as shown in Fig. 1(d). Starting from these source points, the Voronoi regions grow, with their growth limited to connected areas as shown in Fig. 1(e). This constraint ensures that perfusion territories are assigned within anatomically connected regions.

Assuming that arteries branch and supply blood to their perfusion territories, the model estimates the flow rate at the truncated point, representing total flow to the downstream vasculature within that territory. The blood flow rate required to perfuse each region is calculated by multiplying the mass of the assigned territory by the cerebral blood flow (CBF), which is the blood flow required per unit mass of tissue, as follows:

$$\:{Q}_{i}=\rho\:\sum_{j=1}^{{n}_{i}}{v}_{j}\cdot CB{F}_{j}$$

where $\:{Q}_{i}$ is the total blood flow rate (mL/min) required to perfuse the tissue elements assigned to the $\:i$th source point, $\:{n}_{i}$ is the number of the assigned elements, and $\:\rho\:$ is the density of the tissue element. The volume and CBF value of the $\:j$th tissue element are represented by $\:{v}_{j}$, and$\:\:CB{F}_{j}$, respectively. In this study, the tissue density was assumed to be constant of 1.04 g/mL^28^ and different CBF values were applied depending on the region of the brain tissue. The gray and white matter of the cerebellum and cerebrum were assumed to be 0.8 mL/min/g and 0.2 mL/min/g, respectively^29^ while the rest of the tissue was assumed to be uniformly 0.5 mL/min/g^30^. Tissue types were classified based on the segmentation obtained from FreeSurfer. Furthermore, the study investigated the influence of regional CBF variations by comparing the results with those obtained using a uniform CBF value of 0.5 mL/min/g in all regions.

Finally, a one-dimensional blood flow simulation was performed to calculate blood flow rates of all cerebrovascular arteries. With the assumptions of a rigid wall and no external force, the Navier-Stokes equations are approximated as the following one-dimensional flow equations:

$$\:Continuity\:equation: \frac{\partial\:Q}{\partial\:z}=0\:\:$$

$$\:Momentum\:balance\:equation:\frac{\partial\:Q}{\partial\:t}+\frac{\partial\:}{\partial\:z}\left[\left(1+{\updelta\:}\right)\frac{{Q}^{2}}{S}\right]+\frac{S}{{\uprho\:}}\frac{\partial\:p}{\partial\:z}=N\frac{Q}{S}+{\upnu\:}\frac{{\partial\:}^{2}Q}{\partial\:{z}^{2}}$$

where $\:z$ is the axial coordinate, $\:Q$ represents the flow rate, $\:p$ denotes the pressure. $\:S$ represents the cross-sectional area, $\:{\uprho\:}$ is the density, $\:{\updelta\:}$ is the velocity profile function, and $\:N$ is the viscous loss parameter. Assuming a parabolic flow profile, $\:\delta\:$ is set to 1/3, and $\:N$ is defined as $\:-8{\uppi\:}{\upnu\:}$, where $\:\nu\:$ is the kinematic viscosity. The density and dynamic viscosity were set at 1.06 g/cm³ and 0.04 g/cm/s, respectively. The continuity equation ensures the conservation of mass, while the momentum balance equation accounts for pressure variations due to blood viscosity, inertia, and vascular geometry along the vessel path. In the simulation, all vessels were assumed to be healthy and cross-sectional area changes within each segment were neglected. Each segment was considered to have a uniform cross-sectional area corresponding to its average value.

The inlet boundary condition of a mean aortic pressure of 93.33 mmHg was assigned to the ICA and BA assuming negligible pressure loss between the aorta and these arteries. The outlet boundary condition was set using the blood flow rate computed from the previous step. For the outlet source points, calculated blood flow values were assigned. For the source points in non-terminal segments, the assigned flow was assumed to bifurcate from the midpoint of the segment by adding a virtual segment originating at the midpoint as demonstrated by sources #2 and #4 in Fig. 1(f).

### Blood flow distribution in the cerebrovascular system

Blood flow distribution was obtained by calculating the relative flow ratios of the left and right middle cerebral arteries (MCA), anterior cerebral arteries (ACA), and posterior cerebral arteries (PCA), and the flow ratios of the left and right internal carotid arteries (ICA) and the basilar artery (BA). The flow rates in the MCA, ACA, and PCA were measured in the M1, A1, and P2 segments, respectively. The flow rates used for this calculation, illustrated in Fig. 1(g), were obtained as described in the previous section.

The flows computed using our proposed methodology were compared to those obtained using Murray’s law, which assumes that the flow rate is proportional to the cube of the radius^11^. The outlet flow rate is calculated as follows:

$$\:{Q}_{i}={Q}_{total}\cdot \frac{{r}_{i}^{3}}{{\sum}_{j=1}^{{n}_{o}}{r}_{j}^{3}},\quad{Q}_{total}=\rho \sum_{k=1}^{{n}_{e}}{v}_{k}\cdot CB{F}_{k}$$

where $\:{n}_{o}$ is the number of outlets, $\:{n}_{e}$ is the number of tissue elements, and $\:{r}_{i}$ is the radius of the $\:i$th outlet. The volume and CBF value of the $\:\text{k}$th tissue element are represented by $\:{v}_{k}$ and $\:CB{F}_{k}$, respectively. The blood flow rate of all non-terminal segments was calculated by solving the one-dimensional blood flow simulation with the same procedures as before. When applying Murray’s law, perfusion through perforating arteries was not considered.

The results were validated with PC-MRI measurements from the literature. Since only average flow rates were reported by these studies^31,32^, the blood flow distribution and their standard deviations were derived by dividing each average flow rate by the sum of the averaged flow rates.

### Perfusion territory and probability map using normalization

Perfusion territory of each artery was estimated by the Voronoi tessellation as shown in Fig. 1(e). Since the flow rate is estimated based on computed perfusion territories, the reliability of these territories was assessed by comparing them with reference atlases. For comparison, the computed perfusion territory of each patient was normalized to the standard template space. In this study, ch2better^33^ and Eva atlas template^34^ were used for the normalization. The registration module of 3D Slicer was used for the registration of the image data and the template^27^. The perfusion territory of each major artery was defined by summing the perfusion territories of its branches. A probability map was created by calculating the probability of perfusing each template space for each major artery as follows:

$$\:{P}_{i}=\frac{{N}_{\overrightarrow{x},i}}{{\sum}_{j\in S}{N}_{\overrightarrow{x},j}}\hspace{1em}\overrightarrow{x}\in X,i\in S \hspace{1em}where \hspace{1em}S=\{MCA, ACA, PCA\}$$

where $\:{N}_{\overrightarrow{x},i}$ is the number of patients where major artery $\:i$ is predicted to perfuse the template coordinate $\:\overrightarrow{x}$. The template space is represented by $\:X\:$and $\:S$ is the set of MCA, ACA, and PCA. Template coordinates were ignored if they were predicted to be perfused by fewer than a predefined number of patients or if they were located outside a mask of the template. The mask was segmented using FreeSurfer^24^with the same regions extracted in the tissue segmentation. Finally, the perfusion territory map was generated by assigning each template coordinate to the major artery with the highest probability.

### Sensitivity study of the physiologic model

**Fig. 2:**
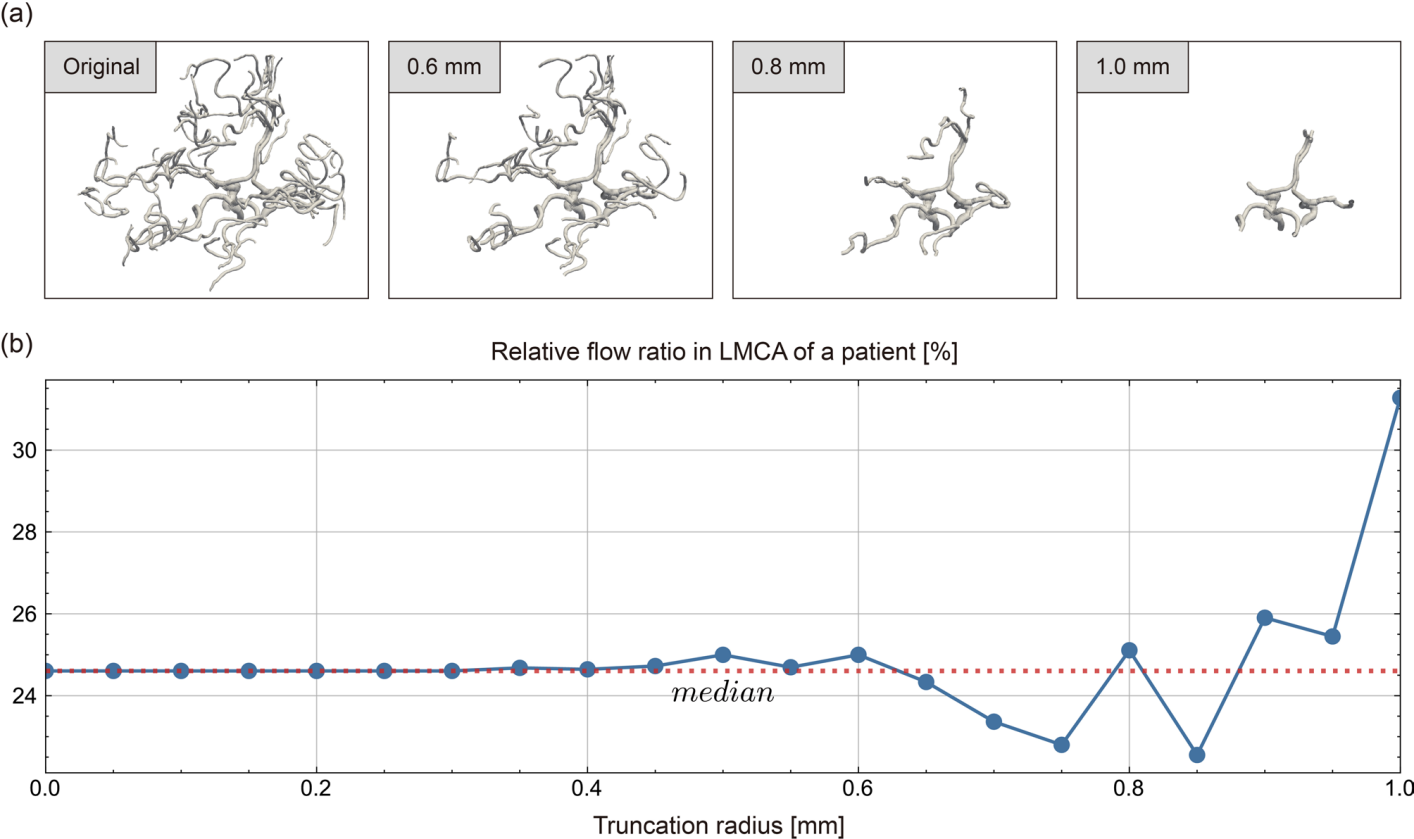
(**a**) Changes in a representative segmented geometry as the truncation radius changes. (**b**) Fluctuation in the relative LMCA flow ratio of a patient as a function of the truncation radius. The median is shown as a red dotted line. LMCA: left middle cerebral artery.

To evaluate the sensitivity of the physiologic model to the changes of the segmented geometry, a sensitivity study was performed by varying a truncation radius of blood vessels. The blood flow distribution of major cerebral arteries was calculated after excluding vessels with a mean radius smaller than the truncation radius and their branches. The truncation radius value was varied from the original segmented geometry up to 1 mm with 0.05 mm increments, resulting in a total of 21 variants for each case. In most patients, only the vessels near the CoW remained when the truncation radius was 1 mm, as illustrated by Fig. 2(a). In the study, the origins of MCA, ACA, PCA, and their parent vessels were constrained not to be removed, and vessels that were not branches or parent vessels of the MCA, ACA, and PCA such as the superior cerebellar artery and ophthalmic artery were excluded from truncation to reduce the influence of vessels known not to perfuse the segmented tissue.

The blood flow distribution may change as the geometry changes as displayed in Fig. 2(b). We define the robustness based on the variability of the blood flow distribution in response to geometric variations. Sensitivity was evaluated using the mean and standard deviation of the difference between the relative flow ratio at each truncation radius and the median for each major artery. The difference from the median was used instead of the mean to avoid the contribution of large fluctuations that may appear when the truncation radius approaches 1 mm.

### Patient data

Data from the MR database of the CASILab at the University of North Carolina at Chapel Hill^35^ and dataset collected at Seoul National University Hospital (SNUH) were used for this study. As the CASILab dataset is de-identified and publicly available, the secondary analysis of the data did not require additional Institutional Review Board (IRB) review. The SNUH dataset was collected in compliance with the Declaration of Helsinki and its subsequent amendments and approved by the Institutional Review Board of Seoul National University Hospital (IRB No. 2305-145-1434). The Institutional Review Board of Seoul National University Hospital waived the requirement for informed consent as the study involved the use of retrospectively collected, de-identified clinical data.

The CASILab dataset contains 3-Tesla T1 MRI and MRA acquisitions from 100 healthy patients. The MR angiographies have a voxel size of 0.5 × 0.5 × 0.8 mm and the T1 MR images have an isotropic voxel size of 1.0 mm. The T1 MR images cover the whole brain, while the MRA images cover the top of the basilar artery (BA) to the tip of the head as shown in Fig. 1(c). Since the vertebral arteries (VA) and the lower part of the BA were not captured in the MRA images, the cerebellum and brainstem were excluded in the tissue segmentation. This study focused on patients aged 18 to 49 years to ensure a comparable study population with the literature data^31,32^ used for comparison. Of the 62 healthy young patients aged 18–49 years, 1 patient was excluded due to poor MRA image and 21 patients were excluded due to the presence of artifacts in the T1 image resulting in poor segmentation of the temporal lobe, leaving a total of 40 subjects included in this study (35 ± 8 years). The patients were labeled with the identifier C, and we utilized identical patient identifiers as in the database.

The SNUH dataset was used for direct validation of the estimated perfusion territories using patient perfusion imaging. Vessel-selective ASL (vs-ASL) was used to capture tissue volumes perfused by labeled vessels. This dataset includes 1.5-Tesla MRA, 3-Tesla T1 images, and vs-ASL images. The patients were labeled with the identifier S to distinguish them from the CASILab dataset. The T1 MRI images have an isotropic voxel size of 1.0 mm and the vs-ASL images were acquired with a voxel size of 1.88 × 1.88 × 6 mm. The MRA image resolution differs for each patient: the MRA image of patient S1 has a voxel size of 0.56 × 0.56 × 0.5 mm, and the MRA image of patient S2 has a voxel size of 0.41 × 0.41 × 0.6 mm.

In contrast to the CASILab dataset, the MRA image of the SNUH did not cover the entire head and therefore many branches of the ACA were not included. Consequently, fewer arteries were segmented for this dataset. Instead, as the intradural vertebral artery was included in the MRA images, the cerebellum and brainstem were not excluded in the tissue segmentation. Patient S1 had a right carotid artery stenosis, which was treated with a stent placement and patient S2 underwent a superficial temporal artery (STA)-to-MCA bypass due to an occlusion in the left distal carotid artery. For both patients, vs-ASL was obtained in the LICA, RICA, and LVA or RVA. The perfusion territory of each patient was estimated using the proposed physiologic model and comparison with vs-ASL was performed after manually aligning the images using landmarks.

## Results

### Blood flow distribution in the cerebrovascular system

The flow rate of all cerebrovascular arteries is estimated by solving the governing flow equations in conjunction with boundary conditions based on the proposed physiologic model and Murray’s law. Blood flow distribution is determined by calculating the flow ratios of the left and right MCAs, ACAs, and PCAs, as well as the flow ratios of the left and right ICAs and BA. The results were compared with those of the literature acquired using PC-MRI by S. Amin-Hanjani et al.^31^ and L. Zarrinkoob et al.^32^. In the study of L. Zarrinkoob et al., the flow rate in the PCA was measured at P2, whereas in S. Amin-Hanjani et al., it was measured at P1. Except for the PCA, the flow rates of the major arteries were measured at the same location. To compare our results with similar age groups, we selected the 18–40 years age group from S. Amin-Hanjani et al. (*n* = 122, 31 ± 6 years) and the 20–30 years age group from L. Zarrinkoob et al. (*n* = 49, 25 ± 2 years). The comparison results are shown in Table 1. The table shows the average relative flow ratios and standard deviations for 40 patients. Since L. Zarrinkoob et al. did not present the flow rate separately for the left and right sides, only a single value is shown for the MCA, ACA, PCA, and ICA. It is observed that the average flow ratios calculated by the proposed methodology and Murray’s law fall within one standard deviation of the mean in all major cerebral arteries. The distribution of the estimated flow ratio using the developed model and Murray’s law is reported in Supplementary Figure S1. Supplementary Table S1 presents a comparison of the relative flow ratios in the major cerebral arteries estimated by the proposed methodology using uniform and non-uniform CBF values. The results show that the average flow ratios remained largely unchanged, with the absolute difference between the two approaches being less than 0.2% of the average flow ratio.

**Table 1:** Comparison of the blood flow distribution estimated by the proposed methodology (denoted by “Tessellation”) and murray’s law with literature data obtained by PC-MRI measurements (mean ± std [%]). The study includes 40 subjects aged 18–49 years (35 ± 8), while S. Amin-Hanjani et al.^31^ studied 122 subjects aged 18–40 years (31 ± 6) and L. Zarrinkoob et al.^32^ investigated 49 subjects aged 20–30 years (25 ± 2).

	Tessellation	Murray’s law	PC-MRI measurements (S. Amin-Hanjani^31^)	PC-MRI measurements (L. Zarrinkoob^32^)
LMCA	24.4 ± 1.5	24.8 ± 4.2	25.6 ± 5.0	26.2 ± 5.0
RMCA	24.2 ± 1.6	25.7 ± 3.5	24.0 ± 4.7	
LACA	13.7 ± 5.1	14.4 ± 5.6	14.2 ± 3.9	14.3 ± 2.9
RACA	15.3 ± 5.1	15.5 ± 5.4	14.5 ± 4.8	
LPCA	11.2 ± 1.6	9.4 ± 2.9	10.9 ± 2.1	9.4 ± 2.1
RPCA	11.1 ± 2.0	10.2 ± 3.0	10.9 ± 2.4	
LICA	37.9 ± 5.7	37.5 ± 6.4	39.6 ± 6.7	38.7 ± 6.6
RICA	41.6 ± 6.7	41.0 ± 7.5	38.8 ± 7.5	
BA	20.5 ± 4.8	21.5 ± 5.2	21.6 ± 6.4	22.7 ± 6.2

### Perfusion territory and probability map using normalization

**Fig. 3:**
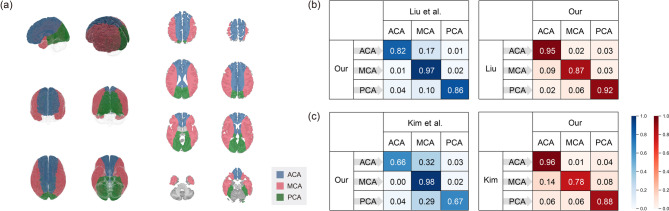
(**a**) Perfusion territory map of the major cerebral arteries estimated by the proposed methodology. Comparison with the atlas estimated by (**b**) Liu et al.^36^ and (**c**) Kim et al.^37^ The left tables show the percentage of voxels classified as ACA, MCA, or PCA according to the proposed methodology among the voxels assigned to each of these territories by the atlases and vice versa for the right tables.

We compared the estimated perfusion territories with those of the literature data. Each patient’s cerebral perfusion territories are estimated by the proposed methodology and are normalized to the standard template. Template coordinates predicted to be perfused by fewer than 10 out of 40 patients were excluded from the perfusion territory map. The estimated perfusion territory map of the major cerebral arteries is illustrated in Fig. 3(a). The calculated perfusion probability maps for the two template spaces are provided in Supplementary Figure S2.

The results are compared with those of the reference atlases obtained by mapping cerebral infarct regions to the major cerebral arteries. Figure 3(b) shows a quantitative comparison with the atlas estimated by Liu et al.^36^. For the voxels classified in the region of ACA, MCA, and PCA in our map, the left table calculates how many of them were classified in the region of ACA, MCA, and PCA in Liu et al. and vice versa, for the right table. The probability that voxels classified as ACA, MCA, and PCA in our map are the same in Liu et al. is 82%, 97%, and 86%, respectively. This comparison excludes the region of the VA, as the MRA images used in this study did not cover the VA and the lower part of the BA.

Additional comparison was made with the atlas estimated by Kim et al.^37^. Since the atlas is not available to the public, Kim et al. conducted a comparison study of their atlas with our map using the same criteria and provided us with the results (Fig. 3(c)). The probability that voxels classified as ACA, MCA, and PCA in our map are the same in Kim et al. is 66%, 98%, and 67%, respectively. It is observed that 32% of the voxels classified in the territory of ACA and 29% of the voxels classified in the territory of PCA are classified in the territory of MCA in the atlas of Kim et al.

### Subject-specific blood flow distribution and perfusion territory estimated by the supply and demand relationship

**Fig. 4:**
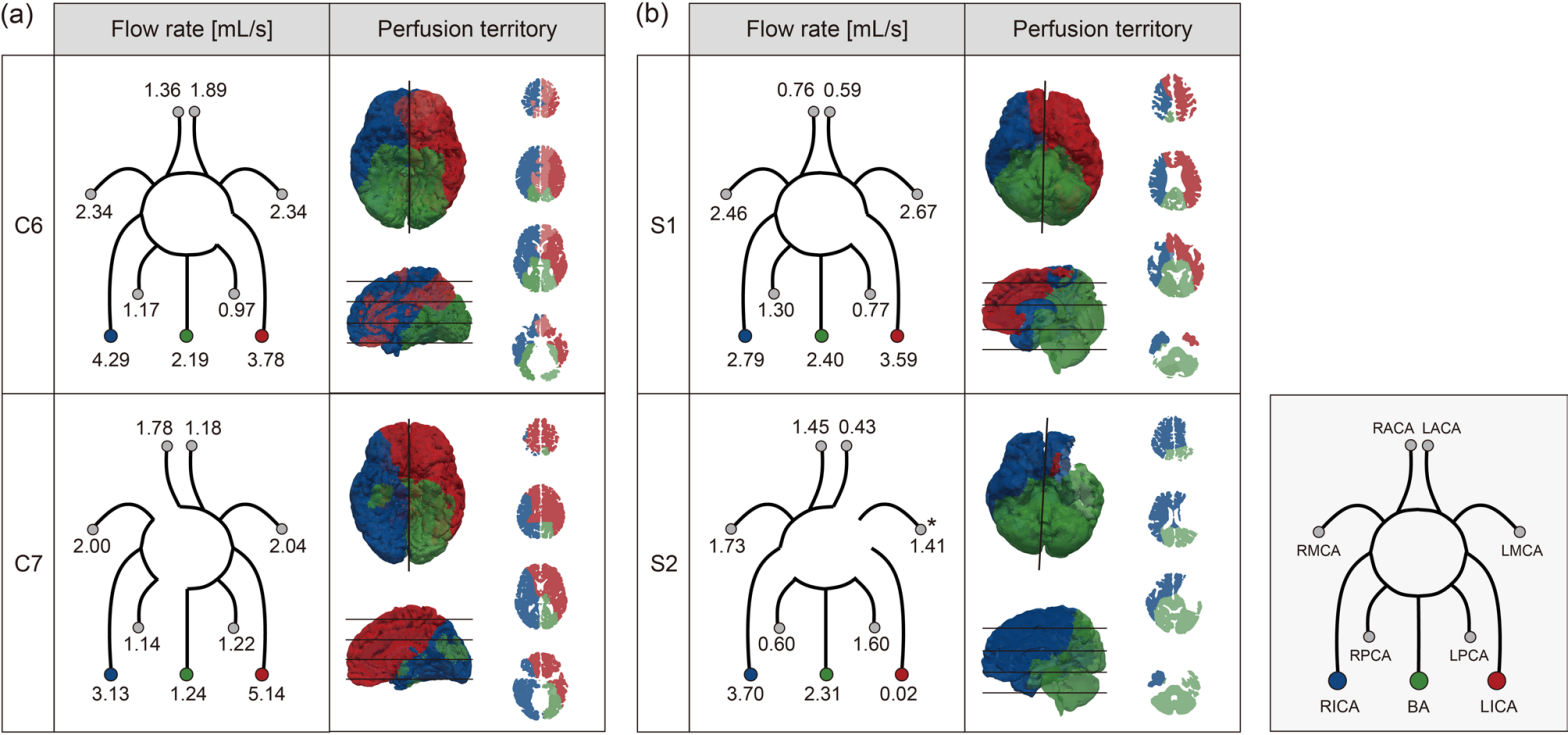
Estimated flow rate and perfusion territory of (**a**) two patients from the CASILab dataset and (**b**) two patients from the SNUH dataset. The regions perfused by multiple major cerebral arteries are visualized using overlapping layers with different colors and opacities, reflecting the proportional contribution of each artery. The asterisk (*) in the LMCA of Patient S2 indicates the region is likely supplied by the external carotid artery.

The flow rates and perfusion territories of each patient were estimated by applying the proposed methodology for the patient data of the CASILab. The results of two patients are displayed in Fig. 4(a) for illustrative purposes. Regions perfused by multiple major arteries simultaneously are represented by overlapping layers with different colors and opacities based on the proportion supplied by each major artery. Patient C6 in the figure has all the communicating arteries except the left posterior communicating artery. It is observed that some of the blood inflow from the RICA travels to the LACA. Patient C7 in the figure has an anatomical variation in which the RICA is not connected to the ACA and the BA is not connected to the RPCA.

### Comparison of perfusion territories with vessel-selective ASL

**Fig. 5:**
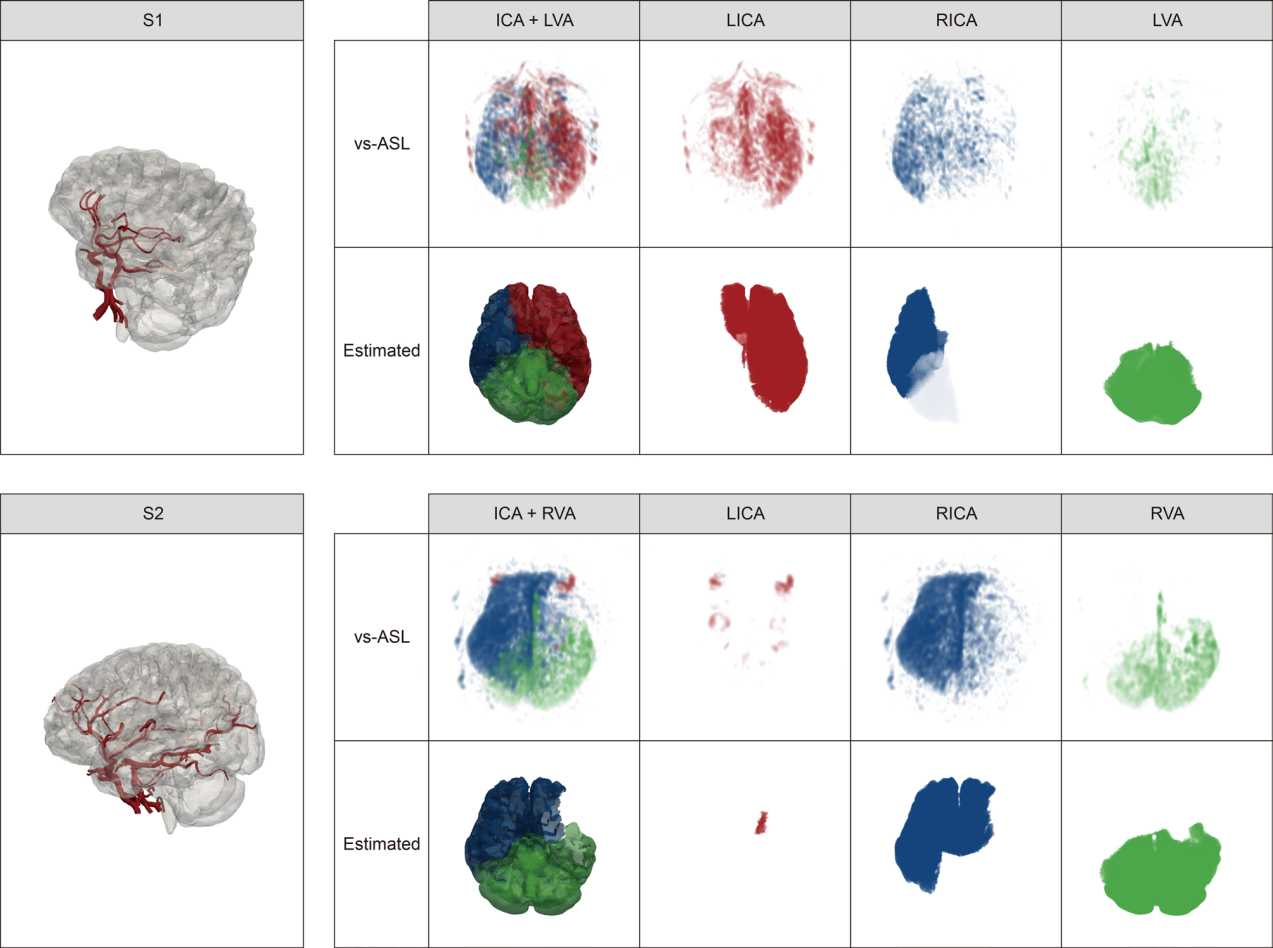
Comparison of estimated perfusion territory with vs-ASL of the patients from the SNUH dataset. The color intensity in regions supplied by multiple arteries is adjusted to be inversely proportional to the contribution of each artery. vs-ASL: vessel-selective arterial spin labeling.

For direct comparison with perfusion imaging, the proposed model was applied to two patients from the SNUH dataset. As illustrated in Fig. 4(b), Patient S1 had all the communicating arteries except the left posterior communicating artery. In the case of patient S2, it was observed on the MRA image that the LMCA was disconnected from the ICA and BA, and the ACA was not connected to the LICA. The right posterior communicating artery was not present. The asterisk (*) marked in the LMCA of Patient S2 indicates the region likely to be supplied by the external carotid artery. This is due to the fact that Patient S2 had undergone an STA-MCA bypass surgery, which may allow the external carotid artery to supply blood to the marked region through the bypass graft. The segmented geometries of the patients and the comparison of the estimated perfusion territory with vs-ASL are displayed in Fig. 5. In regions supplied by multiple major arteries, color intensity fades inversely according to the contribution ratio of each artery.

### Sensitivity study of the physiologic model

**Fig. 6:**
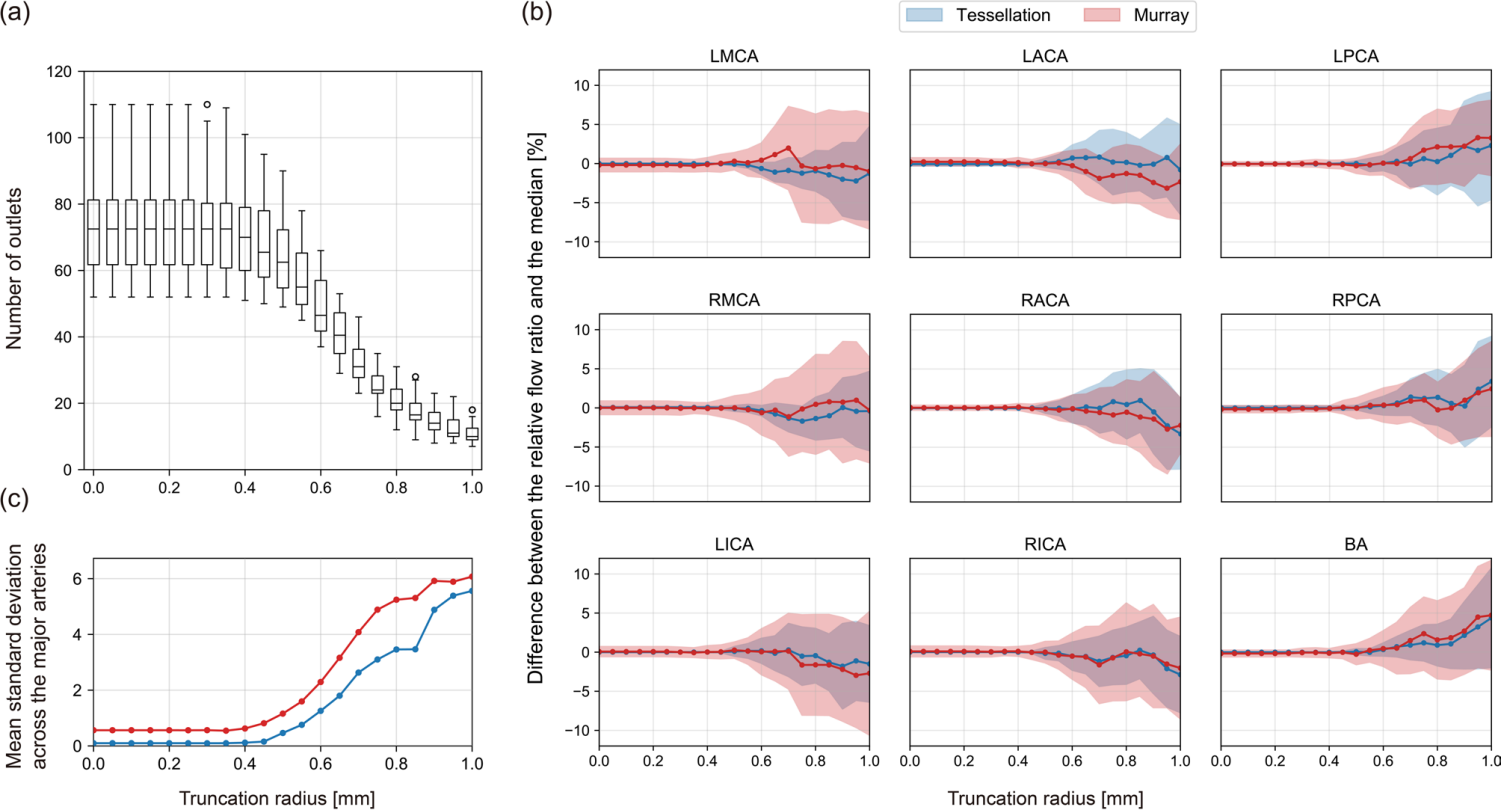
(**a**) Change in the number of outlet segments as the truncation radius changes. The circles represent outliers. (**b**) The difference between the relative flow ratio for each truncation radius and the median. The line represents the mean difference and the shaded area indicates its standard deviation. (**c**) The mean standard deviation across all major arteries.

The robustness to geometric changes is evaluated by investigating the dependence of flow ratios on outlet vessel truncation radius values for the proposed methodology and Murray’s law. As the truncation radius value increases, the number of outlet vessels decreases rapidly, especially at about 0.4 mm and only the vessels near the CoW remain at 1.0 mm as shown in Fig. 6(a). Figure 6(b) reports the mean and standard deviation of the difference, calculated for each patient, between the relative flow ratio for each truncation radius and the median for each major artery. In the figure, the line represents the mean of the differences and the shaded area represents its standard deviation.

## Discussion

### Blood flow distribution and perfusion territory

We have shown that the proposed physiologic model can predict blood flows and perfusion territories of major cerebrovascular arteries for each patient using medical image data only. The model estimates perfusion territories of the arteries by associating them with a subject-specific tissue geometry and calculates flow rates accordingly. The cerebrovascular system is known to have many anatomical variations with collaterals and perforating arteries but the proposed model estimates perfusion territories and flow rates of the cerebrovascular system reasonably when comparing against population-averaged values.

Blood flow distribution of the major cerebral arteries calculated using the developed model was compared against those using Murray’s law. Table 1 shows that the computed blood flow distribution using the developed model is realistic when compared against the literature data. The results of Murray’s law are also in good agreement with the literature, likely due to the fact that the vessel segmentation was carried out very thoroughly in this work, resulting in a large number of vessel outlets with a comparable size. However, while the averages were similar when examining individual cases, we found that there were often discrepancies between the relative ratios calculated using the developed model and Murray’s Law.

Additionally, as shown in Supplementary Table S1, there was little difference in the relative flow ratios of the major cerebral arteries when estimated using uniform versus non-uniform CBF values. The reason for the little difference is due to the fact that the regions supplied by the major cerebral arteries are large enough such that the differentiation of the white and gray matter does not make a big difference. However, the difference in flow ratios between uniform and non-uniform CBF values becomes significant as the radius of the cerebrovascular artery decreases.

We compared the perfusion territory map estimated by the proposed methodology with those of the literature atlases. As shown in Fig. 3(b), the computed perfusion territory map is in good agreement with that of Liu et al. However, the perfusion territory map does not agree well with that of Kim et al. This is because, according to Fig. 3(c), a number of voxels classified as regions of ACA and PCA in the estimated map were predicted to be regions of MCA in the atlas of Kim et al. Such discrepancy was also reported by Liu et al. When they compared their map to that of Kim et al., they found a good agreement in most areas except for the regions in the superior occipital gyri and the superior and middle frontal orbital regions. They confidently classified the majority of the superior occipital gyri regions as PCA regions due to the larger amount of PCA infarct data compared to those of Kim et al. They also argued that classifying a large portion of the superior and middle frontal orbital regions as ACA was more consistent with other literature, but they acknowledged that there are limitations due to the small number of ACA infarct data. As Liu et al. noted, infarcts occur most often in the MCA and rarely in the ACA, which may lead to imprecise perfusion boundaries. Moreover, the poor agreement may be due to the fact that the characteristics of the patients in this study were different from those of Kim et al. with the presence of disease.

Although the estimated perfusion territory map did not agree well with that of Kim et al., the map agreed well with that of Liu et al., and noticeable similarity is observed when the estimated perfusion territories are directly compared to the vs-ASL (Fig. 5). We validated the estimated perfusion territories with vs-ASL for the two diseased patients. We expected lower accuracy as the MRA images did not cover the entire brain. Many branches of the ACA were missed in the segmented cerebrovascular arteries and only a small number of vessels were extracted. However, as shown in Fig. 5, the estimated perfusion territory of the LICA and RICA showed fair agreement with those of the vs-ASL in patient S1. There was also no significant difference in the estimated perfusion territory of the VA compared to that of the vs-ASL. In the case of patient S2, as the LICA was not connected to other major vessels, few perfusion territories appeared in both the prediction and the vs-ASL as displayed in the figure. Additionally, the predicted perfusion territory of the RICA was largely in agreement with that of the vs-ASL. Note that only the RICA was predicted to perfuse these regions because some of the vessels supplying the left frontal and parietal lobes were not extracted from the MRA images. Lastly, contrary to population-averaged perfusion atlases, the RVA was predicted to supply blood to the left side of the brain and similar perfusion territories were reported from the vs-ASL.

Even though the comparison was conducted for the two patients only, these results demonstrate that the developed model may be applied to predict perfusion territories of major cerebral arteries. This aligns with the findings in Fig. 4(a), demonstrating the model’s capability to effectively account for personalized patient characteristics. Further, the results indicate that the proposed model predicts cerebral perfusion territories and may be used to predict possible infarct regions and the severity when some vessels are diseased. Conversely, when a patient presents with stroke symptoms, the affected perfusion territory may provide clues on the vessels affecting the ischemia. The association between the cerebral arteries and tissues and the prediction of perfusion territories, however, warrants more validation in the future.

Previously, diseased perfusion territories have been estimated by means of imaging modalities such as perfusion imaging or diffusion-weighted imaging^36–40^. While these imaging modalities are effective in estimating the perfusion territory of diseased vessels, they have shown limitations when measuring infarcts due to relatively small blood vessels other than major cerebral arteries. As an alternative approach, computational methods have been reported to estimate perfusion territories using more extensive cerebrovascular arteries. Linninger et al. and Rundfeldt et al. utilized synthetic tree generation algorithms to predict perfusion territories by constructing extensive cerebrovascular arteries^41,42^. Although these methods can estimate perfusion territories of various vessel radii, they require the construction of the synthetic vessel networks to associate the precapillary or arteriole vessels with the tissues. Padmos et al. estimated perfusion territories of cerebral arteries based on the outlet flows calculated using Murray’s law^43^. This method can be implemented without the construction of the synthetic vessel networks. However, since their approach is based on Murray’s law, it is highly sensitive to the radii and number of segmented vessels. Compared to the existing methods, the proposed methodology provides a unified, coherent workflow which computes blood flow and the associated perfusion territory simultaneously. Additionally, it does not require the construction of synthetic vessel networks beyond the medical image resolution.

Further, we investigated variations in flow distribution for healthy patients. Figure 4(a) shows the results of two patients with different anatomical characteristics as an illustration. For subject C6, blood flow through the anterior communicating artery is reasonably predicted which indicates that the model can assess the function of the primary collateral circulation, which could help to assess a disease severity. It is also seen that the ACA perfuses the contralateral cerebrum in this patient, meaning a diseased LACA could cause infarction in the right cerebrum. In subject C7, only the LICA supplies blood to the perfused region of the ACA and only the RICA supplies blood to the perfused region of the RPCA, showing different perfusion territories than those of subject C6. The flow rate of the LICA is greater than that of the RICA as the perfusion territory volume perfused by the LICA is larger. The results show that flow rates and perfusion territories depend on the patient geometry and that the proposed physiologic model is able to account for subject-specific characteristics. Further, the model calculates total inflow using the subject’s tissue geometry extracted from medical images. This allows us to predict inflow in a more personalized way than reported studies which often rely on population-averaged quantities.

### Robustness of the physiologic model

In order to assess the sensitivity of the developed model to the segmentation uncertainties, a sensitivity study was performed by truncating outlet vessels with a gradually increasing truncation radius value. Figure 6(b) shows that there is no significant difference in ACA and PCA for both the proposed model and Murray’s law, but the flow ratios calculated using the developed model exhibit less fluctuations to the changes of the segmented geometry in MCA, ICA, and BA compared to those of Murray’s law. These results can be explained by the increased robustness of the developed model with regard to the segmentation variations in vessel radius compared to Murray’s law. The reason why Murray’s law is more sensitive to changes in the truncation radius is due to the nonlinear dependency on the vessel radius and the number of vessels. The results of the developed model, however, show a rapid deviation from the median when the truncation radius is greater than 0.8 mm, likely because only the vessels near the CoW remain, no longer faithfully representing the actual cerebrovascular geometry. Based on the results of the sensitivity study, it is recommended to segment all vessels down to a radius of 0.7 mm when using the developed model and down to 0.5 mm when using Murray’s law. In particular, when using the proposed model, it may be suggested that the major cerebral arteries of MCA, ACA, and PCA be modeled far enough to include at least one major bifurcation in the downstream of the circle of Willis. As the side branches tend to have smaller radius values, they may not influence the physiologic model significantly and may be omitted.

### Physiologic model for boundary conditions

As the proposed physiologic model estimates cerebral blood flows and perfusion territories realistically when compared against population-averaged data and is more robust to the truncation radius values, the estimated blood flow values using the physiologic model may serve as boundary conditions for the cerebrovascular models. The flows may serve as Dirichlet boundary conditions for relatively healthy subjects as the assigned flows are assumed to be supplied by the cerebral arteries with little pressure loss in the arterial system. When applying to diseased patients, however, we suggest that we assign as Neumann boundary conditions using lumped parameter models so that the flow for each cerebral artery is computed given the actual patient geometry and the boundary conditions.

### Limitations

There are several limitations in this study. First, the flow rate estimated by the developed model was not validated with direct measurements from the same patients. Instead, the validation was performed using average flow ratios from the literature data, which may not fully represent the individual variability. Validation using transcranial color-coded Doppler is going to be performed in a future study. Second, the perfusion territory map was generated by normalizing the map to a standard template and compared to those of the literature atlases, but a small number of literature atlases was available for comparison and the characteristics of the compared subjects were different. The estimated perfusion territories were compared with those of vs-ASL to address this limitation, but the number of patients compared using vs-ASL was too limited and a quantitative comparison was not made due to the low resolution of the vs-ASL. More thorough comparison study will be conducted to quantitatively validate estimated perfusion territories. Furthermore, while this study did not validate the model using flow rate measurements and perfusion imaging data from the same patients, future studies will utilize both data types to refine the physiologic model, thereby improving model accuracy. Third, the perfusion territory of smaller arteries other than major cerebral arteries was not validated. Further comparisons with perfusion imaging are planned in a follow-up study. Fourth, the sensitivity of the developed model to MRI quality, such as resolution and artifacts, has not been investigated. While obtaining vessel centerlines from medical imaging is generally less sensitive to imaging quality compared to determining vessel radius values, the vessel centerlines can still be influenced by the quality of the MRI. Furthermore, the developed model relies not only on the centerlines of the segmented vessel geometry but also the segmented tissue geometry. If medical imaging fails to adequately capture certain regions, it may lead to inaccurate estimates of the flow rate of the vessels supplying those regions. Future studies will explore the effect of MRI quality and field of views (FOV) on the developed model using a larger number of images obtained from standard MRI imaging protocols. Lastly, the effect of the secondary collateral circulation was not considered in the proposed methods. The recruitment of secondary collateral circulation may lead to discrepancies between the actual flow and the flow estimated by the developed model. Future work will extend the methods to account for the secondary collateral circulation and to study its effects on the cerebrovascular system.

## Conclusion

In this study we propose a physiologic model of the cerebrovascular system based on a supply and demand relationship between arteries and tissues. The model constructs both the cerebrovascular arteries and brain tissues from input MRI image data and computes an association map between them using the Voronoi tessellation methods. The proposed model was validated using the datasets of 40 healthy young subjects and two diseased patients. For the dataset of 40 healthy young subjects, the computed blood flow distribution is realistic when compared against that of the literature data. The model reasonably predicts perfusion territories of major cerebral arteries when compared to those of the literature atlases. Additionally, the computed perfusion territories of the two diseased patients were compared against those of vs-ASL and they showed reasonable agreement. Lastly the proposed model is shown to be more robust to the changes in number of vessels and vessel caliber of a segmented geometry compared to Murray’s law. These results indicate that the proposed physiologic model may be used to predict subject-specific cerebral arterial blood flows and perfusion territories using medical image data only. This model may serve as an alternative to Murray’s law in estimating blood flows of the cerebrovascular system and assigning boundary conditions for blood flow simulations of the cerebrovascular system not to mention that the model may identify possible infarct regions and the severity.

## Electronic supplementary material

Below is the link to the electronic supplementary material.

Supplementary Material 1

## Data Availability

The data generated and/or analyzed during this study are available from the corresponding author upon request.
