# Supplementary material for: Physiologic model of the cerebrovascular system using supply and demand between arteries and tissues
Source: Sci Rep. 2025 Jul 30;15:27785. doi: 10.1038/s41598-025-10223-7 (PMC12310993; doi:10.1038/s41598-025-10223-7)

**Supplementary Figure S1**: The calculated flow ratios of the left and right MCA, ACA, and PCA and flow ratios of the left and right ICA, and BA of 40 healthy patients from the CASILab dataset. The dashed lines within each violin indicate the quartiles of the distribution.


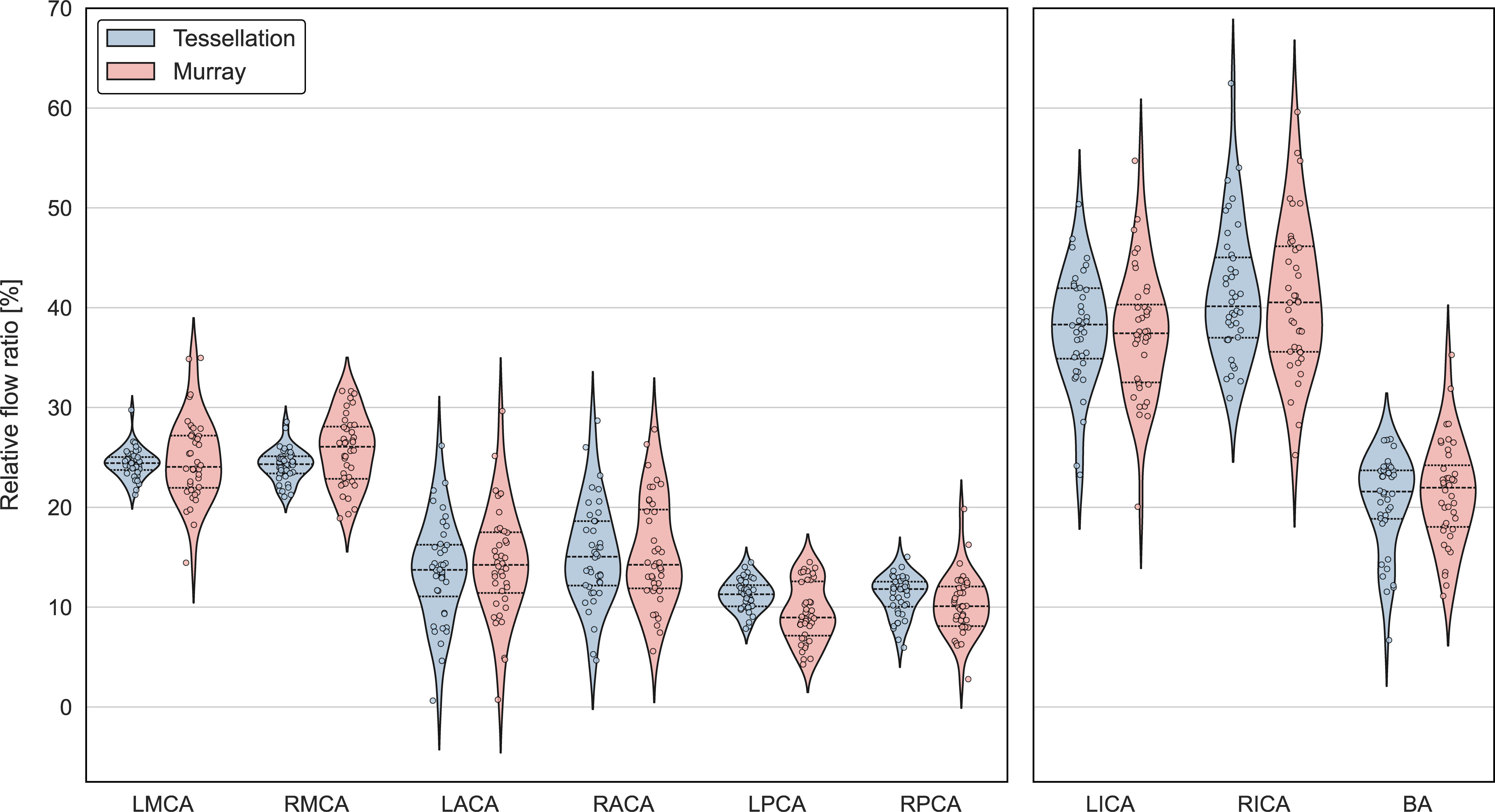


**Supplementary Table S1:** Comparison of the relative flow ratios estimated by the proposed methodology using uniform and non-uniform CBF values (mean ± std [%]). CBF: cerebral blood flow.

|  | Uniform CBF | Non-uniform CBF |
| --- | --- | --- |
| LMCA | 24.2 ± 1.6 | 24.4 ± 1.5 |
| RMCA | 24.1 ± 1.7 | 24.2 ± 1.6 |
| LACA | 13.8 ± 5.3 | 13.7 ± 5.1 |
| RACA | 15.5 ± 5.2 | 15.3 ± 5.1 |
| LPCA | 11.2 ± 1.4 | 11.2 ± 1.6 |
| RPCA | 11.1 ± 1.8 | 11.1 ± 2.0 |
| LICA | 37.8 ± 5.7 | 37.9 ± 5.7 |
| RICA | 41.7 ± 6.8 | 41.6 ± 6.7 |
| BA | 20.4 ± 4.8 | 20.5 ± 4.8 |

**Supplementary Figure S2:** Perfusion probability maps for major cerebral arteries of two template spaces. The color intensity represents the probability of perfusion for each major artery within the template spaces, calculated according to the equation presented in Section 2.5. Lower probabilities are represented with increased transparency.

- ch2better template


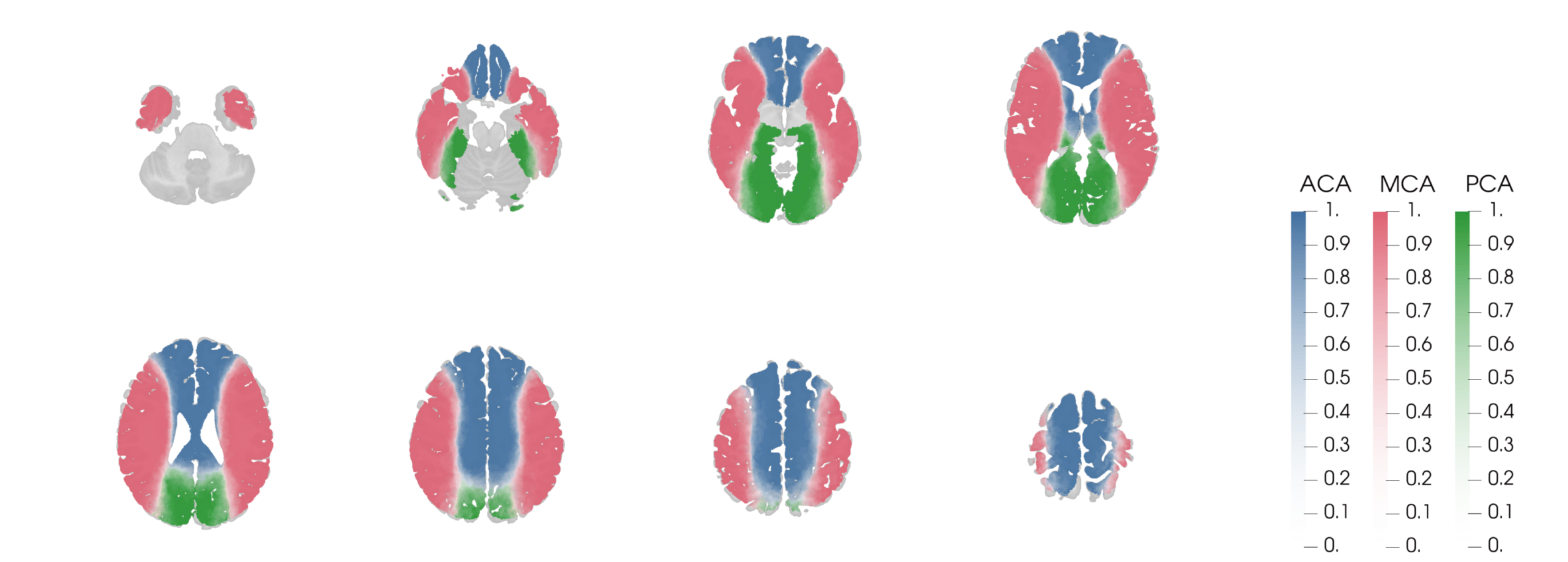


- Eva atlas template


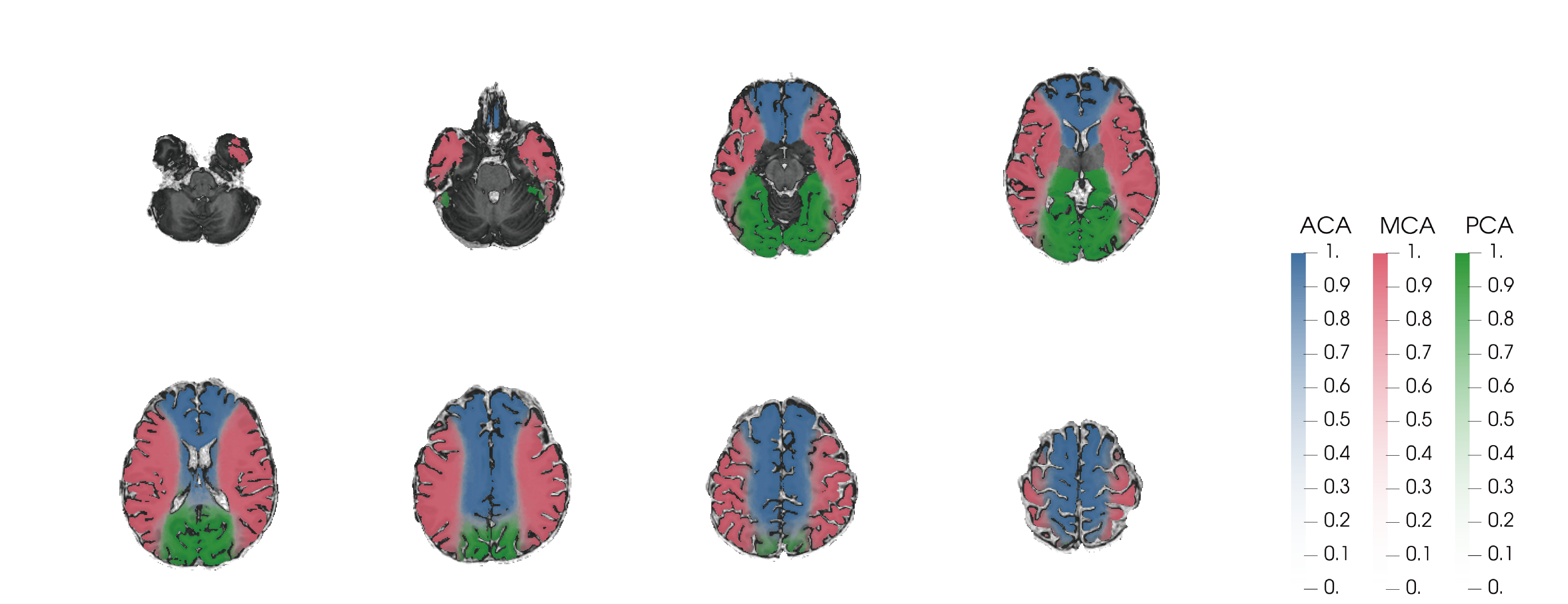

Supplement: Supplementary file 1 — Supplementary Material 1 [file 41598_2025_10223_MOESM1_ESM.docx]
